# Cognitive behavioural treatment for the chronic post-traumatic headache patient: a randomized controlled trial

**DOI:** 10.1186/1129-2377-15-81

**Published:** 2014-12-02

**Authors:** Dorte Kjeldgaard, Hysse B Forchhammer, Thomas W Teasdale, Rigmor H Jensen

**Affiliations:** 1 Danish Headache Center, Department of Neurology, Glostrup Hospital, University of Copenhagen, Copenhagen, DK, Denmark; 2 Department of Neurology, Glostrup Hospital, University of Copenhagen, Copenhagen, DK, Denmark; 3 The Department of Psychology, Faculty of Social Sciences, University of Copenhagen, Copenhagen, DK, Denmark; 4 Danish Headache Center, Department of Neurology, Glostrup Hospital, University of Copenhagen, Nordre Ringvej 69, DK-2600 Glostrup, Copenhagen, DK, Denmark

**Keywords:** Chronic post-traumatic headache, Treatment, Cognitive therapy, Brain concussion, RCT

## Abstract

**Background:**

Chronic post-traumatic headache (CPTH) after mild head injury can be difficult to manage. Research is scarce and successful interventions are lacking.

To evaluate the effect of a group-based Cognitive Behavioural Therapy (CBT) intervention in relation to headache, pain perception, psychological symptoms and quality of life in patients with CPTH.

**Methods:**

Ninety patients with CPTH according to ICHD-2 criteria were enrolled from the Danish Headache Center into a randomized, controlled trial. Patients were randomly assigned to either a waiting list group or to a nine-week CBT group intervention. At baseline and after 26 weeks all patients completed the Rivermead Post Concussion Symptoms Questionnaire, SF-36, SCL-90-R and a headache diary.

**Results:**

The CBT had no effect on headache and pressure pain thresholds and only a minor impact on the CPTH patients’ quality of life, psychological distress, and the overall experience of symptoms. The waiting-list group experienced no change in headache but, opposed to the treatment group, a significant decrease in somatic and cognitive symptoms indicating a spontaneous remission over time.

**Conclusions:**

Our primarily negative findings confirm that management of patients with CPTH still remains a considerable challenge. Psychological group therapy with CBT might be effective in an earlier stage of CPTH and in less severely affected patients but our findings strongly underline the need for randomized controlled studies to test the efficacy of psychological therapy.

## Background

Post-traumatic headache (PTH) is one of the most frequent symptoms following mild to moderate head injury and is a cardinal symptom in the definition of the post-concussional syndrome, but reports of the incidence of PTH after injury to the head differs widely between 30% and 90%, probably because of different diagnostic criteria and study methodologies [1]. Furthermore, the incidence and prevalence of chronic post-traumatic headache (CPTH) is uncertain. CPTH is defined as a secondary headache in the International Classification of Headache Disorders (ICHD-2) [2] (Table 1). The classification has been revised in 2013, adding a more detailed description of the traumatic injury to the head causing the CPTH [3]. Research studies into the epidemiology of CPTH are few, despite the fact that CPTH is very costly for both the individual patient and society in general. In the Akerhus population-based study of chronic headache among 30–44 year-old persons, the prevalence of chronic post-traumatic headache (CPTH) after mild head injury was reported to be 0.15% in men and 0.20% in women [4]. Also the aetiology is still debated [5]. The lack of correspondence between severity of the action of external forces on the head and persistence of symptoms has led to the assumption that psychological factors may play a crucial role in the cause and maintenance of CPTH [6].

**Table 1:** Diagnostic criteria of chronic post-traumatic headache (CPTH) attributed to mild head injury (5.2.2) according to ICHD-2

**A.**	Headache, no typical characteristics known, fulfilling criteria C and D
**B.**	Head trauma with all the following:
	1. either no loss of consciousness, or loss of consciousness of <30 minutes’ duration
	2. Glasgow Coma Scale (GCS) ≥13
	3. symptoms and/or signs diagnostic of concussion
**C.**	Headache develops within 7 days after head trauma
**D.**	Headache persists for >3 months after head trauma

Treatment studies of CPTH are likewise scarce and in most reported studies only small changes in headache frequency, duration, or intensity have been reported after treatment [7]. A prior study from Zeeberg et al. [8] of the efficacy of the multidisciplinary treatment in the Danish Headache Center (DHC) showed that all diagnostic groups with the exception of the CPTH group benefitted from the treatment.

The pharmacological treatment of CPTH in general relies on treatment guidelines for primary headache [9]. Randomized, prospective, double-blind treatment trials of PTH are scarce [10] and the outcome of pharmacological intervention is modest in the clinic [11].

There are a number of different types of non-pharmacological approaches to treatment including biofeedback, relaxation training, physical therapy, counselling, psychotherapy e.g. cognitive behavioural therapy (CBT) individually and in groups.

A review by Lew et al. [12] identified only five studies on non-pharmacological treatment published between 1990 and 2005. Watanabe et al. [7] found a few more studies but none of these could be classified above American Academy of Neurology class III criteria for evidence. Previous clinical trials on the effect of psychological intervention, have not furnished strong evidence as a basis for the treatment of CPTH and the studies [13] have methodological shortcomings. They use different definitions of traumatic injury to the head and PTH is not systematically characterized according to ICHD classification. Sample sizes are often small (1–40 patients) and there is a lack of control groups. In the study by Gurr et al. [14] a combination of CBT and relaxation training have shown promising results. Intervention based on the cognitive model, involves a collaborative formulation of patients’ emotional, physiological, and behavioural responses mediated by their perceptions of experience [15]. The effect of this treatment has been documented for a number of chronic disease groups in addition to headache patients [16], but studies involving a controlled design are lacking.

The present study is part of a larger clinical CPTH investigation [17,18]. The aim of the study reported here has been to conduct a randomized controlled trial (RCT) exploring whether a group-based CBT intervention would lead to a relative decrease in headache, pain perception and psychological symptoms and an increase in quality of life in the study group compared to a waiting-list control group. Because the field of CPTH treatments is so understudied the present study has also aimed to describe and generate hypotheses for the future research.

## Methods

### Participants

Patients with CPTH who were referred to the Danish Headache Center (DHC), a multidisciplinary, tertiary headache centre [8] were evaluated. Headache specialists at the centre referred patients to the research psychologist and first author (DK) as part of their treatment program. The study was conducted between June 2008 and August 2011.

Eligible participants were patients who were diagnosed with CPTH attributed to mild head injury according to ICHD-II [2] (Table 1) and who were interested in psychological headache treatment. Included patients were adults aged 18–65 years without other neurological or psychiatric disorders. Patients were excluded on basis of information from their medical record complemented by interviewing the patients about other neurological or psychiatric disorders. We did not perform any additional tests in order to validate this information.

Exclusion criteria were pregnancy, present medication and/or substance overuse as defined by ICHD-II, a history of pre-existing primary headache defined as more than 12 days of tension-type headache annually and/or more than one migraine attack per month in the preceding year as well as prior psychological treatment in DHC. Patients who had developed CPTH in relation to a whiplash injury were also excluded. When there was doubt as to whether the injury included neck trauma, the patient was requested to describe the traumatic injury to the head in greater detail, in order to exclude an acceleration/deceleration trauma. Patients were also excluded if neuroimaging (MR/CT-scan) showed signs of contusions or other traumatic brain lesions.

**Figure 1:**
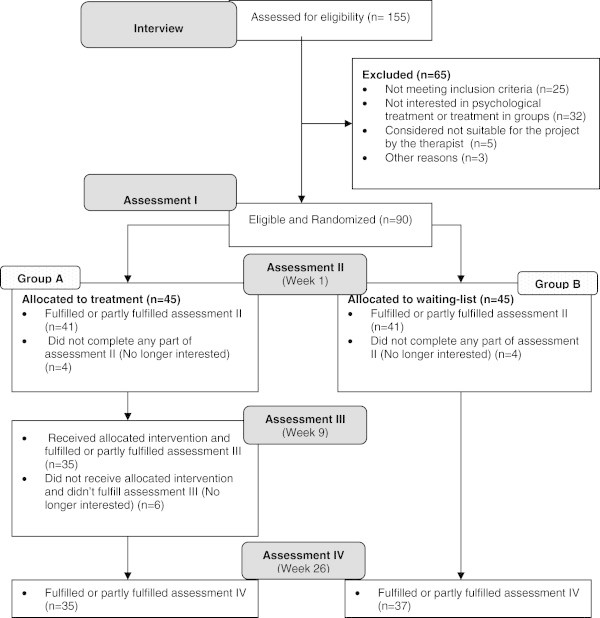
Flow-diagram of chronic post-traumatic (CPTH) patients assessed, included and treated in DHC.

The patients were evaluated for inclusion by means of a structured interview about current headache, general demographic features and information about the trauma to the head. Patients, who were eligible, and who wanted to participate in the trial, were required to agree not to engage in physiotherapy treatment in the DHC and to keep their pharmacological treatment stable throughout the 26 weeks the trial took place. The patients were, however, free to contact neurological and other medical consultations within and outside the centre. After the interview, eligible patients were asked to complete the Harvard Trauma Questionnaire (HTQ) and the NEO-PI-R personality profile, the results of which have been presented in previous publications [17,18]. The questionnaires were sent to the patients by mail with a request to return them in a prepaid envelope (Assessment I) (Figure 1). Patients who did not return the questionnaires after two reminders were excluded. Allocation to either the treatment group (Group A) or to the waiting-list group (Group B) was based on a computer generated randomization and took place when the questionnaires from assessment I were received in a still-sealed envelope. The patient was then informed about the allocation.

Treatment patients were informed about the first treatment session where also assessment II took place. Waiting-list patients were also invited to an assessment II where their pressure pain thresholds were recorded and the questionnaires (Rivermead Post Concussion Symptoms Questionnaire, SCL-90-R, SF-36 and a headache diary) handed out. Patients were instructed to complete them at home and again return in prepaid envelope. Assessment III for the treatment group included the questionnaires RPQ and SF-36 as well as a measure of pressure pain threshold. After 22 weeks both groups were again sent the same questionnaires as at Assessment II, together with a date to consult the researcher (DK) where the questionnaires should be handed in and where a final evaluation for both groups was completed (Week 26).

The study followed the guidelines for trials of behavioural treatment for recurrent headache [19]. All participants gave their written informed consent according to the Declaration of Helsinki. The study was approved by the regional Ethics Committee (H-1-2011-FSP) and by the Danish Data Protection Agency.

### Interventions

The intervention was based on the principles of Beck [15] and all sessions followed the same format inspired by Gurr [14] and Mittenberg [20].

The theoretical basis for our applied treatment model relies on Jacobson’s [21] multi-factorial model. In the model biological, social, cognitive, affective and behavioural factors are integrated in order to explain the development and maintenance of the post-concussional syndrome. Based on cognitive-behavioural theory the model provide an theoretical frame for understanding how individual patients’ beliefs, appraisals and coping responses directly influence the development and maintenance of symptoms over time.

Our focus was to educate and to provide strategies that enabled the patients to explore and understand their situation and encouraged them to focus on possible changes in their lives (primarily changing cognitive assumptions) that over time might lead to a more active everyday life (better quality of life and less psychological stress). Relaxation techniques in the form of Autogenic Training (AT) was also used because a positive effect has been documented in the treatment of tension-type headache and migraine [22]. AT is a self-relaxation procedure by which a psycho- physiological determined relaxation response is elicited. In addition, progressive muscle relaxation (a technique for reducing tension by alternately tensing and relaxing the muscles), breathing techniques and guided visualization were applied [23].

Each weekly session included the following: 1) review and discussion of the material and assignment from the previous session 2) instruction, discussion and practice of new material 3) instruction and practice of new relaxation techniques (session 3–8) 4) presentation of the following week’s assignment. Materials and assignments relevant to the current week’s topic were handed out each session. On session 3 the patients received a CD with the relaxation techniques including one technique per session. Participants were encouraged to attend all sessions and to complete their assignments. A more detailed description of the content of each session is shown in Table 2.

**Table 2:** The objectives of each CBT session for the CPTH treatment patients

**Session**	
1	Introduction to the group and the diagnosis CPTH and it’s possible origins.
2	Introduction to the cognitive model and the stress-pain connection, identify stressors, and goal setting.
3	What is concentration? Memory problems and their possible origin when having headache. Relaxation technique: breathing exercise.
4	Memory and reading strategies. Management of energy. Relaxation technique: Progressive muscle relaxation.
5	Pain model. Acceptance and behaviour towards headache. Relaxation technique: short breathing exercise with body scan.
6	Acceptance of present headache state. Management of energy. Relaxation technique: visualisation of pleasant place.
7	Define and identify Negative Automatic Thoughts (NAT) Relaxation technique: visualisation mental preparation.
8	Examine NAT, develop alternative more adaptive thoughts. Relaxation technique: visualisation problem solving.
9	Integration and maintenance of new techniques and concepts.

The treatment was delivered in a group format with eight CPTH patients in nine weekly sessions (two hours each). All the groups were led by the first author, a licensed clinical psychologist who had prior and extensive experience of treating patients with headache with CBT therapy. Groups were started when eight consecutive patients had been assigned to the treatment group.

The waiting-list Group B patients did not receive any active treatment within the DHC for the 26 weeks during which they participated in the study, but were promised that they would receive active psychological treatment after the 26 weeks.

### Outcomes

#### Headache assessment

Headache was recorded in a four-week baseline period at the beginning of Assessment II and at the end of Assessment IV (Figure 1) via a basic headache diary [24] which has been validated [25] and which records the duration and intensity of any headache on a daily basis. In the case of missing values the patients were interviewed in detail about frequency, duration and intensity of their headaches through the preceding four weeks. The primary outcome variable was the area-under-the-headache curve (AUC) [25] and calculated as the sum of the daily recordings of headache duration multiplied by headache intensity. The clinical characteristics of the headache assessment have been published elsewhere [18].

#### Pressure pain thresholds

Pressure pain threshold was defined as the pressure at which the sensation changed from pressure alone to a combination of pressure and pain. It was measured at the dorsum of the second finger (middle phalanx) using a hand-held pressure algometer (Somedic AB, Sweden, stimulation probe 0.5 cm^2^, pressure loading rate of 22 kPa/s) [26].

#### Health-related quality of life

Health-related quality of life was measured with the Danish version of the SF-36 [27]. This consists of 36 questions concerning perceived health-related quality of life, with answers being divided into eight scales measuring health concepts: physical functioning (PF), role physical (RP), role emotional (RE), social functioning (SF), bodily pain (BP), mental health (MH), vitality (VT) and general health (GH). On each scale the score ranged from 0–100, with a high score indicating better perceived health [28,29].

#### Concussion symptoms

The Rivermead Post Concussion Symptoms Questionnaire (RPQ) [30] is a validated questionnaire [31] that measures the severity and number of cognitive, emotional and somatic symptoms commonly experienced after concussion. A more detailed description of our use of the questionnaire is presented elsewhere [18].

#### Psychological distress

The Symptom Checklist (SCL-90-R) is a validated 90-items questionnaire of general mental distress and includes nine primary scales: somatisation, interpersonal sensitivity, anxiety, psychoticism, depression, hostility, paranoid ideation, phobic anxiety and obsession-compulsion. Items are scored on a 5-point Likert scale (0 = not at all, to 4 = extremely) according to how much discomfort the symptoms caused during the preceding week [32]. Raw scores were converted to T-scores, using Danish norms, for each of the nine primary scales and a Global Severity Index (GSI) scale prior to data analysis. Caseness was defined as a T-score on the GSI ≥63. In this study a slightly modified version of the Symptom Checklist SCL-90-R containing an additional two items (SCL-92) was used.

### Outcome measures

The primary endpoint was the relative change in score on the RPQ, SCL-90-R, SF-36, Headache diary and Pain pressure thresholds between Assessment II and Assessment IV after 26 weeks in both groups. The secondary endpoints were differences on the same measures between treatment and waiting-list group 26 weeks after baseline.

### Sample size and effect size

We have calculated the effect size of group differences by using Cohen’s ‘d’ which expresses the magnitude of the difference between two group means in terms of standard deviation units. Thus two group means differing by half of a (common) standard deviation would represent a Cohen’s ‘d’ of .5. Using this statistic as a criterion we have predicted the difference between pre- and post-treatment for each measure (headache, pain perception, psychological symptoms and quality of life) to represent an effect size (Cohen's d) of 0.4. The clinically relevant difference was set to 0.3 between groups [33]. A risk of 5% for type I errors and 20% for type II errors was accepted. The number of subjects therefore needed in each group was estimated to be 30. Anticipating the risk of dropout rate, we aimed to include at least 35 patients in each group [34].

### Randomisation

The study was designed as a parallel-group study with balanced allocation ratio (1:1). The random allocation sequences were in blocks of ten patients conducted using computer-generated lists administered by a colleague from DHC not involved in the study. Neither the research therapist DK nor the patients themselves could be blinded throughout the study, but collected data were not viewed until the patients had either finished the 26-weeks study period or had dropped out of the study.

At inclusion, all patients had been informed about the likelihood that they would be assigned to the waiting-list condition and informed about the withholding of treatment for 26 weeks. After allocation patients in the waiting-list group were promised that they would receive the same psychological treatment as the active treatment group after 26 weeks if they were interested.

### Statistical methods

The data were analyzed using SPSS version 19.0. Average values of continuous variables are described by means and standard deviations (SD). Categorical variables are described by frequencies and percentages. Chi-square analysis was used in the case of categorical variables and for frequency analyses. McNemar's test was used for the marginal frequencies of binary outcomes of occupation (before/after trauma).

Pre- and post-treatment measures were compared separately for the treated and waiting-list groups using matched paired t-tests. We also conducted direct comparisons of the two groups at post-treatment time. However, despite the randomization, there were some differences between the treated group and the waiting-list group on several measures at the pre-treatment time. We have therefore adopted analyses of covariance to test for group differences in post-treatment outcome measures co-varying for the corresponding measures taken pre-treatment.

In all cases p < 0.05 (two-tailed) was considered to be statistically significant.

## Results

A flow diagram of the 155 patients assessed for the treatment study can be seen in Figure 1. Ninety patients were included and in summary the sample comprised 56% women, with a mean age of 34 years (SD = 11.3). More than half of the participants were married (54%) and the majority (39%) were educated on the level of skilled labour. Before the trauma 89% were employed full-time after the trauma only 22% were employed fulltime. The trauma was often caused by traffic accidents (45%) and the mean duration since the injury was 27 months. Further descriptive data has been described in a prior publication by Kjeldgaard et al. [18]. No statistically significant differences in the clinical characteristics between patients in Group A and B were identified.Of the total sample of enrolled patients (N = 90), four patients assigned to group A, and four patients in group B never completed assessment II because they declined treatment or dropped out subsequently. In Group A, 35 patients fulfilled or partly fulfilled assessment IV versus 37 patients in group B (Figure 1). In Group A, 33 patients (89%) attended 6–9 sessions, and four patients (11%) attended 3–5 sessions.

### Headache

No significant change in headache activity (frequency, intensity and headache index) or in terms of Pressure Pain Thresholds after the treatment (Table 3) could be identified in Group A or Group B compared to the pre-treatment baseline values. When adjusted for the small and non-significant differences in terms of headache activity between Group A and B at assessment II (baseline), this difference did not influence the headache activity outcome.

**Table 3:** Headache and pressure pain thresholds for the treatment (A) and waiting-list (B) group of chronic post-traumatic headache (CPTH) patients

	**Group A (N = 34)**					Group B (N = 33-37*)					
	***Assessment II***		***Assessment IV***			***Assessment II***		***Assessment IV***			***p *** **††**
					**d †**					**d †**	
Frequency (days/ 4 weeks)	26.4	(4.39)	26.0	(5.13)	.067	25.9	(4.87)	25.1	(6.51)	.144	.551
Intensity (0–10 scale)	6.33	(1.78)	6.34	(1.69)	.007	5.45	(1.79)	5.10	(2.27)	.172	.128
Headache index (intensity x duration)	2341	(1110)	2261	(1140)	.072	1880	(1157)	1772	(1288)	.088	.621
Pain threshold	221	(96.4)	215	(98.7)	.067	217	(78.4)	211	(100)	.065	.979

Data are presented as mean (SD).

*Number varies due to missing values.

†Cohen’s d, for within group differences.

††Analysis of covariance, group comparison of assessment IV measures controlling for assessment II measures.

### Quality of life

Regarding Quality of Life (QoL) as measured by the SF-36, all scores for Group A were increased at post treatment evaluation, indicating slightly better QoL although the improvement was not significantly different from Assessment II. Group B also experienced slightly better scores on all SF-36 sub scales (except General Health) after 6 months waiting time. On the scales of Role physical (p = .005) and Bodily pain (p = .027) Group B had improved significantly (Table 4). When adjusted for the differences between Groups A and B at Assessment II, Group B only improved significantly on the Bodily pain scale (p = .020) at assessment IV.

**Table 4:** SF-36 for the treatment (A) and waiting-list (B) group of chronic post-traumatic headache (CPTH) patients

	**Group A (N = 35)**					**Group B (N = 37)**					
	***Assessment II***		***Assessment IV***			***Assessment II***		***Assessment IV***			**p ††**
					**d†**					**d †**	
Physical function	68.6	(18.0)	70.7	(15.7)	.127	72.3	(19.4)	75.4	(18.4)	.165	.434
Role physical	8.57	(21.0)	14.3	(22.1)	.266	12.8	(23.3)	26.4	(32.2)	.480	.107
Bodily pain	25.9	(17.4)	26.3	(14.4)	.025	29.5	(18.6)	37.7	(22.8)	.396	.020
General health	51.7	(19.8)	52.5	(20.8)	.035	52.4	(22.5)	49.5	(19.7)	.137	.355
Vitality	27.9	(19.0)	31.0	(21.3)	.156	36.2	(18.6)	40.4	(23.1)	.200	.480
Social function	35.8	(24.3)	44.1	(24.0)	.347	47.0	(26.3)	53.7	(29.4)	.242	.588
Role emotional	68.6	(41.2)	69.5	(38.2)	.024	59.5	(42.4)	63.0	(45.0)	.082	.776
Mental health	52.7	(17.4)	56.6	(20.0)	.207	61.4	(17.9)	65.1	(19.3)	.197	.716

Data are presented as mean T-scores (SD).

†Cohen’s d, for within group differences.

††Analysis of covariance, group comparison of assessment IV measures controlling for assessment II measures.

### Concussive symptoms

The number of symptoms (other than headache) was measured by the RPQ and the somatic, the emotional and the cognitive factors all improved within Group A after the treatment, but also not to a significant level (Table 5). In Group B symptoms also improved but when adjusted for the difference in scores at assessment II between Group A and B, only Group B had significantly less somatic (p = .027) and cognitive symptoms (p = .039) compared to baseline values.

**Table 5:** Rivermead for the treatment (A) and waiting-list (B) group of chronic post-traumatic headache (CPTH) patients

	**Group A (N = 34)**					**Group B (N = 37)**					
	***Assessment II***		***Assessment IV***			***Assessment II***		***Assessment IV***			**p ††**
					**d†**					**d †**	
Sum total	33.4	(13.8)	32.0	(12.6)	.107	30.0	(13.4)	25.7	(12.8)	.327	.054
Cognitive factor	2.49	(1.47)	2.63	(1.43)	.095	2.50	(1.47)	2.16	(1.42)	.237	.039
Emotional factor	1.96	(1.37)	1.64	(1.05)	.265	1.48	(1.07)	1.25	(1.02)	.220	.550
Somatic factor	2.01	(.817)	1.95	(.672)	.079	1.83	(.822)	1.57	(.754)	.331	.027

Data are presented as mean T-scores (SD).

†Cohen’s d, for within group differences.

††Analysis of covariance, group comparison of assessment VI measures controlling for assessment II measures.

### Psychological distress

The results on the SCL-90-R (Table 6), measuring psychological distress level, did in fact show that Group A gained some degree of psychological improvement after treatment. On the subscales regarding Interpersonal sensitivity (p = .015), depression (p = .030) and hostility (p = .003) we identified a significant effect towards better outcome after active treatment. Group B only improved in terms of phobic anxiety (p = .028) while being on the waiting-list, whereas there was no such change on this scale in Group A. When adjusted for the difference scores at assessment II between Groups A and B, Group A only tended to improve on the factor Interpersonal sensitivity (p = .050). Neither groups reached the Caseness score on the GSI (≥63) at any time.

**Table 6:** SCL-90-R for the treatment (A) and waiting-list (B) group of chronic post-traumatic headache (CPTH) patients

	**Group A (N = 34)**					**Group B (N = 36)**					
	***Assessment II***		***Assessment IV***			***Assessment II***		***Assessment IV***			**p ††**
					**d†**					**d †**	
Somatisation	62.2	(7.19)	61.5	(7.72)	.099	59.3	(8.08)	58.7	(7.77)	.092	.545
Obsession-compulsion	61.2	(10.8)	59.4	(9.95)	.179	58.4	(10.2)	57.1	(10.6)	.126	.966
Interpersonal sensitivity	55.7	(9.45)	52.7	(11.1)	.300	50.2	(13.1)	51.1	(12.2)	.074	.050
Depression	61.4	(9.36)	58.7	(9.77)	.279	57.5	(8.80)	55.3	(10.0)	.233	.804
Anxiety	60.0	(8.45)	57.8	(11.0)	.223	55.3	(10.4)	53.5	(11.1)	.168	.982
Hostility	60.8	(7.63)	56.4	(10.0)	.505	54.4	(10.3)	54.3	(10.6)	.016	.363
Phobic anxiety	59.2	(12.5)	56.5	(12.8)	.214	58.2	(12.5)	55.2	(13.2)	.235	.790
Paranoid ideation	48.7	(11.8)	48.0	(10.5)	.066	50.0	(10.9)	48.3	(11.1)	.151	.642
Psychoticism	54.0	(12.4)	51.8	(11.7)	.179	52.2	(10.5)	51.5	(11.0)	.064	.588
Global severity index	60.7	(8.15)	59.3	(8.89)	.164	57.2	(9.16)	55.7	(9.79)	.153	.734

Data are presented as mean T-scores (SD).

†Cohen’s d, for within group differences.

††Analysis of covariance, group comparison of assessment IV measures controlling for assessment II measures.

## Discussion

The present CBT intervention had only a minor impact on the CPTH patients’ quality of life, psychological distress, symptoms overall and had no effect on the headache itself. In contrast, we found fewer somatic and cognitive symptoms in the waiting-list group, suggesting a spontaneous remission over time.

### Headache

Overall the CPTH patients did not experience any change in headache activity (intensity, duration and headache index) or in terms of pain perception measured by Pressure Pain Thresholds over time. We had expected that psychological group treatment would have had a positive influence on the headache and pain parameters but we were unable to detect any changes and not even what might have been a placebo response. We consider this to be probably due to a fairly high degree of chronicity and a very long duration of the headache problem in these patients. The majority of our patients had already tried multiple pharmacological as well as non-pharmacological treatments without adequate effect so these CPTH patients did suffer from a particularly refractory type of headache. Whether an earlier intervention, completed a shorter time after the traumatic event where patients presumably would be less chronic, would have been more effective in regard to the alleviation of headache and pain perception remains an issue for future research.

### Quality of life

We had also expected an improved quality of life as measured by the SF-36 because the intervention focused on some of the issues measured by the SF-36. Looking at the results all scores in Group A are raised from pre- to post-treatment, indicating slightly better QoL but the improvement was not significant. Education regarding the overall acceptance and how physical activity might reduce such symptoms and the risk of depression was also introduced in the treatment. That could be part of the reason that the factor “Psychical Role” tended to improve. Feeling socially isolated because of the headache was quite often discussed by the patients in the treatment sessions. The scores on “Social function” improved also to a higher level but there was still no significant change between pre- and post- treatment assessment. After 26 weeks on the waiting-list, Group B, by contrast, increased their QoL on the “Bodily pain” factor to a significant level, which suggests a spontaneous and minor remission in pain, a finding that, however, not could be observed in the treatment group.

### Concussion symptoms

Although the patients did not experience less headache after the treatment, all items on the RPQ did improve in Group A after treatment, although not significantly.

CPTH is often part of a more complex postconcussional syndrome [13] including a variety of both cognitive and other somatic problems. These various symptoms were not specifically addressed in the treatment session, where in particular the overcoming of emotional problems (e.g. feeling depressed and frustration) was in focus. The waiting-list group experienced less somatic and cognitive symptoms at assessment IV which in terms of somatic symptoms may parallel their decrease in “Bodily pain” on the SF-36.

### Psychological distress

The results on the SCL-90-R measuring psychological distress level show that Group A, but not Group B, improved in terms of interpersonal sensitivity, depression, hostility and anxiety. The improvement after treatment in regard to interpersonal sensitivity might reflect a higher level of self-awareness and insight into the relationship between headache symptoms and behavioural limitations. The lowered level of hostility in the treatment group might also be seen as an indicator for an increased level of self-awareness and a more realistic perception of psychological and bodily signals.

A lower hostility score might also reflect patients after treatment experiencing less anger towards themselves (e.g. “why can’t I just get my act together?”) and/or towards others (e.g. “why does nobody understand my situation?”). Greater insight into individual psychological mechanisms does not always lead directly to measurable changes in behaviour and it may be difficult to identify in the chosen instruments of the present study. Thus, the trends we have identified here might also become more apparent as a change in behaviour over a longer period of time.

On the other hand neither groups at any time reached the Caseness score on the GSI (≥63)*.* This could both be due to the exclusion of patients with psychiatric comorbidity but also that psychological distress is not specific to CPTH patients [17].

Overall, the intervention did not succeed in reducing symptoms or increasing QoL to a significant and clinically relevant level. The treatment group, however, did show slightly better results, primarily in terms of decreased psychological distress although this decrease did not match that reported by Gurr et al. [14].

### Methodological considerations

A number of factors might explain our relatively limited results. When the treatment program was developed we had expected a group of severely affected CPTH-patients but almost a third of the CPTH group had a score equal to or above the clinical cut-off score for Post Traumatic Stress Disorder (PTSD) according to the Harvard Trauma Questionnaire (HTQ) [18]. We chose to include the HTQ and these patients to further describe the direction of potential psychological stress that could be a part of CPTH. In retrospect, more effort should have been put into either addressing the PTSD symptoms before/during the treatment or in the pre-treatment screening leading to exclusion or a more equal allocation of patients with PTSD symptoms for this specific treatment program. A post hoc analysis revealed namely that the allocation of patients with PTSD symptoms according to the HTQ were not equal for Group A (38%) and Group B (24%). In treatment Group A patients having high levels of PTSD symptoms prior to treatment had a lower QoL, more symptoms overall and experienced more psychological distress compared to patients without PTSD symptoms [18]. However the non- PTSD patients from Group A revealed no significant improvement after treatment either but an overall exclusion of patients with a high level of PTSD symptoms from the study might have improved the treatment outcome. Further analysis of premorbid personality and level of psychological distress have been discussed in previous papers by Kjeldgaard et al. [17,18].

Another methodological consideration in our treatment approach was the number of sessions offered and the fact that the treatment was only given in a group format. Looking at other mainly uncontrolled treatment studies between 8 and 50 sessions (both individual and in groups) were given. Our nine sessions is thus at the lower end of this range. We offered relatively few sessions because of geographical, financial and practical constraints, but compared to experiences of generally good results of CBT given to patients with primary headaches [16] we had expected to detect a clinically relevant outcome using our chosen compromise. Many patients commented, however, that there were too few treatment sessions; often they felt that they had ‘just got started’ when it was time to finish the group after nine weeks.

In addition, individualized sessions in addition to group treatment were often requested by the patients. However, a relevant balance between available resources and outcome must always be struck and the fact that we identified a slightly better outcome in the waiting-list group is interesting. Further controlled studies are clearly needed to confirm or to contradict these observations.

An advantage of our study was the number of patients included. To our knowledge this is the largest psychological CPTH treatment study to date. Also the use of a control waiting-list group, added methodological value and challenges the existing psychological strategies. Yet another advantage of our study was that our patients were clearly classified as only having CPTH with no other primary or secondary headaches including Medication Overuse Headache (MOH). From our recent study of patients with MOH [35] we know that the majority of these patients with a primary underlying headache, such as migraine or tension-type headache, will experience a 50% or more reduction in their headache after detoxification. Within the latest and more favourable treatment studies of CPTH only Tatrow et al. [36] has reported having controlled for coexisting MOH in her CPTH patients. Gurr et al. [14] did not report whether they controlled for MOH when including patients in their study, and 8 out of their 13 patients had changed their medication during their CBT treatment program. None of our patients had MOH at time of inclusion and the strict exclusion criteria may partly explain why our results were so negative compared to other studies. Another difference from other CPTH-studies is our exclusion of patients with pre-existing headache. The majority of the studies [14,37] did not state explicitly whether or not they included patients with pre-existing headache. Ham [38] did not exclude patients with pre-existing headache and did not state the exact number of patients included with pre-existing headache nor their type of primary headache. In Hickling’s study [39] it is described that none reported a history of headache prior to the trauma, but no more detail is provided. If earlier studies have included patients with pre-existing headache it is not certain that it is actually the CPTH that has decreased through treatment; it may just as well be the pre-existing primary headache.

Owing to chance factors, the treatment and the waiting-list groups were, prior to treatment, homogenous in terms of demographic variables but heterogeneous regarding headache frequency, intensity and headache index as well as on their additional disabilities measured by the RPQ, SCL-90-R and SF-36. On the majority of these assessment parameters the treatment group had higher pre-treatment scores that indicated that they were in fact initially more disabled by the CPTH than the waiting-list group, so all results were controlled for these differences.

The appropriateness of the applied methodology using a waiting list group as controls can always be debated but we consider it to be extremely important to have a control group in all types of headache management although the conventional procedure of using of ‘placebo group’ is difficult if not impossible in non-pharmacological management. De Groot et al. [40] conducted a systematic review of tension type headache patients who had received non-pharmacological treatment, including CBT. They found that patients being on a waiting-list for active treatment had a mean recovery rate of 17.9% (in the literature often reported as a 50% or more improvement on the headache index). In studies where the control condition involved no treatment or just self-monitoring of the headache, the recovery rate was only 6.4% [40].

A spontaneous recovery might explain why the waiting-list group in our study both at assessment II and IV had reduced psychological distress (SCL-90-R), fewer symptoms (RPQ) and better QoL (SF-36), whereas the lack of such remission in the treatment group may be due to a paradox effect of increased awareness of symptoms and disability during the group sessions.

Because the samples of CPTH patients were drawn from a specialised headache centre where patients with relatively chronic, rare and difficult-to-treat headache types are seen, the present results may not be representative for patients with less burdensome CPTH. Likewise, it could be of interest to replicate the study within earlier stages of CPTH.

## Conclusion

Within a randomized and controlled study design we could not identify any improvement of headache in patients with CPTH after group-based psychological CBT management. Although we found some minor positive improvements on psychological symptoms, we were not able to replicate positive QoL outcomes and/or overall reductions of psychological symptoms from the prior, mostly uncontrolled, studies. Surprisingly the waiting list group had a better outcome than the CBT treated group. Although the outcome is minor, there may be a spontaneous remission of CPTH over time. Psychological group therapy with CBT might be effective in an earlier stage of CPTH and in less severely affected patients but our findings underline the need for randomized controlled studies to test the efficacy of existing treatment strategies.

## Abbreviations

PTH: Post-traumatic headache; CPTH: Chronic post-traumatic headache; CBT: Cognitive behavioural therapy; ICHD-2: International classification of headache disorders; DHC: Danish headache center; HTQ: Harvard trauma questionnaire; AT: Autogenic training; RPQ: Rivermead post Concussion symptoms questionnaire; SCL-90-R: Symptom checklist; AUC: Area-under-the-headache curve; QoL: Quality of life; PTSD: Post traumatic stress disorder.

## Competing interests

The research received grants from Lundbeck (R67-A6507) and the Danish “Helsefonden” (2099B033).

DK received honoraria and travel funding from Allergan and Pfizer. HBF, TWT and RHJ have not received any funding relating to this study. RHJ is a board member of EHMTIC, EHF, LTB, ATI, Linde Gas and Neurocore.

## Authors’ contributions

DK, HBF and RHJ conceived the study, and participated in its design and coordination. DK included the patients, conducted the study and drafted the manuscript. TWT supervised the statistical analyses. HBF, RHJ and TWT revised the manuscript critically. All authors read and approved the final manuscript.
